# Fermented Red Ginseng Potentiates Improvement of Metabolic Dysfunction in Metabolic Syndrome Rat Models

**DOI:** 10.3390/nu8060369

**Published:** 2016-06-16

**Authors:** Min Chul Kho, Yun Jung Lee, Ji Hun Park, Hye Yoom Kim, Jung Joo Yoon, You Mee Ahn, Rui Tan, Min Cheol Park, Jeong Dan Cha, Kyung Min Choi, Dae Gill Kang, Ho Sub Lee

**Affiliations:** 1 College of Oriental Medicine and Professional Graduate School of Oriental Medicine, Wonkwang University, 460 Iksandae-ro, Iksan, Jeonbuk 54538, Korea; shadowzetx@hanmail.net (M.C.K.); shrons@wku.ac.kr (Y.J.L.); hyeyoomc@naver.com (H.Y.K.); morality16@hanmail.net (J.J.Y.); aum2668@naver.com (Y.M.A.); tanrui@hanmail.net (R.T.); 2 Hanbang Body-Fluid Research Center, Wonkwang University, 460 Iksandae-ro, Iksan, Jeonbuk 54538, Korea; jihuncjstk@naver.com; 3 Department of Oriental Medical Ophthalmology & Otolaryngology & Dermatology, College of Oriental Medicine, Wonkwang University, 460 Iksandae-ro, Iksan, Jeonbuk 54538, Korea; shadowzetx@hanmail.net; 4 Department of Oral Microbiology and Institute of Oral Bioscience, Chonbuk National University, Jeonju, Jeonbuk 54896, Korea; joungdan@ijrg.re.kr; 5 Department of Research Development, Institute of Jinan Red Ginseng, Jinan, Jeonbuk 55442, Korea; kyungmc@ijrg.re.kr

**Keywords:** fermented red ginseng, metabolic syndrome, obesity, hyperlipidemia, hypertension

## Abstract

Metabolic syndrome including obesity, dyslipidemia and hypertension is a cluster of risk factors of cardiovascular disease. Fermentation of medicinal herbs improves their pharmacological efficacy. Red ginseng (RG), a widely used traditional herbal medicine, was reported with anti-inflammatory and anti-oxidant activity. Aim in the present study was to investigate that the effects of fermented red ginseng (FRG) on a high-fructose (HF) diet induced metabolic disorders, and those effects were compared to RG and losartan. Animals were divided into four groups: a control group fed a regular diet and tap water, and fructose groups that were fed a 60% high-fructose (HF) diet with/without RG 250 mg/kg/day or FRG 250 mg/kg/day for eight weeks, respectively. Treatment with FRG significantly suppressed the increments of body weight, liver weight, epididymal fat weight and adipocyte size. Moreover, FRG significantly prevented the development of metabolic disturbances such as hyperlipidemia and hypertension. Staining with Oil-red-o demonstrated a marked increase of hepatic accumulation of triglycerides, and this increase was prevented by FRG. FRG ameliorated endothelial dysfunction by downregulation of endothelin-1 (ET-1) and adhesion molecules in the aorta. In addition, FRG induced markedly upregulation of Insulin receptor substrate 1 (IRS-1) and glucose transporter type 4 (Glut4) in the muscle. These results indicate that FRG ameliorates obesity, dyslipidemia, hypertension and fatty liver in HF diet rats. More favorable pharmacological effects on HF diet induced metabolic disorders were observed with FRG, compared to an equal dose of RG. These results showed that the pharmacological activity of RG was enhanced by fermentation. Taken together, fermentated red ginseng might be a beneficial therapeutic approach for metabolic syndrome.

## 1. Introduction

Obesity, hyperinsulinemia, hyperlipidemia, and hypertension, *etc.*, such as variable coexistence diseases are characterized by metabolic syndrome [1]. Patients with various diseases have increased. There are several reasons for that is obesity population by western food, cardiovascular disease by hypertension, atherosclerosis, diabetes from insulin resistance, and so on. Patients with metabolic syndrome, as defined by the NCEP-ATP III (National Cholesterol Education Program Adult Treatment Panel III), simultaneously exhibit three or more of the following characteristics: increased blood pressure, increased waist circumference, decreased high-density lipoprotein (HDL) level, increased triglyceride level and hyperglycemia [2]. In metabolic syndrome, the liver is highly affected by excess dietary nutrients from the intestines and inflammatory adipocytokines from enlarged visceral adipose tissues. Thus, fatty liver is considered as a representative of metabolic syndrome [3,4,5]. 

Fructose is an isomer of glucose with a hydroxyl group on carbon-4 reversed in position. It is promptly absorbed and rapidly metabolized by the liver. Increased consumption of fructose commonly leads to rapid stimulation of lipogenesis and Triglyceride (TG) accumulation, which, in turn, leads to reduced insulin sensitivity and hepatic insulin resistance/glucose intolerance [6,7]. Thus, a high-fructose diet induces a well-characterized metabolic syndrome, generally resulting in hypertension, dyslipidaemia and low levels of HDL-cholesterol [8]. In addition, many recent studies suggest that consumption high fructose may be an important risk factor for the development of fatty liver [9]. Rodents, especially rats, are commonly used as a model to mimic human disease, including metabolic syndrome [10]. Similarly, lots of data suggests that experiments of fructose-diet rats tend to produce some of the changes associated with metabolic syndrome, such as altered lipid metabolism, fatty liver, hypertension, obesity and dyslipidemia [11].

Currently, available pharmacological agents for metabolic disorder have a number of limitations, such as various side effects and high rates of secondary failure. Therefore, the demand has increased from those interested in complementary and alternative approaches, including the use of natural herbs. Especially, natural substances and materials based on traditional medicines are of interest for the prevention or obstruction of diseases related to fatty liver, hypertension, high cholesterol and diabetes [12,13,14].

Red ginseng (RG), which is a famous herb of Korean origin,is produced by steaming and drying fresh and raw ginseng. During the steaming process, red ginseng formation allows for numerous chemical changes such as saponin deformation, amino acid changes, and browning reactions, in order to concentrate the activity principles [15,16]. The pharmacological components of red ginseng include various saponins (such as ginsenoside), non-saponins and amino acids. These components are known to have beneficial anti-inflammatory, anti-oxidant, anti-diabetic and anti-aging effects [17,18,19]. Recently, according to lots of literature, fermentation using microorganisms for the production of more effective compounds has been extensively studied. In particular, the pharmacological effects of new saponin generated by red ginseng fermentation have been reported, and this saponin can be mass produced. Fermented red ginseng (FRG) has displayed 30 types of metabolic factors, including Rb1, Rb2, Rc and Rd. Moreover, several studies have already reported that compound K exhibits anti-cancer, anti-diabetic and elevating immune system effects [20]. In addition, many studies have already reported that fermentation can also increase the effectiveness of pharmacological factors in red ginseng through easier and more effective endogenous absorption via degradation into small molecules, as well as disintegration of their toxicity [21]. In addition, several studies have recently reported that FRG elevates hyperlipidemia and protects pancreatic β-cells from streptozotocin toxicity [22,23]. However, the effect of fermented red ginseng on high fructose (HF) diet animal models has not been yet reported. Therefore, the aim of this study was to investigate and compare the effects of dietary supplied FRG and RG on high fructose diet-induced metabolic syndrome. 

## 2. Materials and Methods

### 2.1. Preparation of Fermented Red Ginseng, Red Ginseng and Losartan

The fermented *Red ginseng* and *Red ginseng* extracts were provided from the Institute of JinAn Red Ginseng, Jinan, Jeonbuk Province, Korea. The losartan was purchased from Sigma-Aldrich (Yongin, Korea). For the fermentation of RG (FRG), a microbial strain, *Lactobacillus plantarum* A KFCC11611P, provided from the Korean Culture Center of Microorganisms (KCCM, Seodaemun-gun, Seoul, South Korea) was used for RG and *Rubuscoreanus* Miq. (RC) fermentation. The microbes were precultured in De Man–Rogosa–Sharpe (MRS) (BD biosciences, Sparks, MD, USA) broth medium for Lactobacillus at 30 °C for 24 h before being used for fermentation. For fermentation, 1 L of 0.05 g/m red ginseng (RG) and 1 L of 0.025 g/mL RG with RC mixture in distilled water was prepared and sterilized. After inoculation with 100 mL of precultured *L. plantarum* A, the RG and mixture solution containing the fermentation microbes was incubated at 35 °C for 10 and 5 days, respectively.

### 2.2. Animal Experiments and Diet

All experimental procedures and animal care were conducted in accordance with the National Institute of Health Guide for the Care and Use of Laboratory Animals and were published by the Institutional Animal Care and Utilization Committee for Medical Science of Wonkwang University (approve code WKU14-105). Seven-week-old male Sprague–Dawley (SD) rats were obtained from Samtako (Osan, Korea). All rats were housed in a room automatically maintained under a controlled 12 h light/dark cycle at 23 ± 2 °C with 45%–55% relative humidity. After acclimatization, animals were divided into 5 groups: a control group fed a regular diet, and fructose groups fed the 60% high-fructose (HF) diet with/without RG 250 mg/kg/day or FRG 250 mg/kg/day or losartan 30 mg/kg/day for 8 weeks, respectively. Both diets were purchased from Research Diet, Inc. (New Brunswick, NJ, USA). All groups received a regular diet and the HF diet, respectively, for 8 weeks. The composition of both diets is listed in Table S1.

### 2.3. Estimation of Blood Pressure

Systolic blood pressure (SBP) of rats in all groups were measured at 1, 2, 5 and 8 weeks of period, respectively. SBP was determined by using non-invasive tail-cuff plethysmogrphy method and recorded with an automatic sphygmotonography (MK2000, Muromachi Kikai, Tokyo, Japan).

### 2.4. Estimation of Oral Glucose Tolerance Tests

The oral glucose tolerance tests (OGTT) were performed 2 days apart at 7 weeks. For the OGTT, rats were deprived of food for 12 h. After the food deprivation period, the basalblood samples were obtained from the tail veins of fully conscious rats and were analyzed using a glucometer (Onetouch^®^ Ultra™, Boston, MA, USA) and Test Strip (Life Scan, Chesterbrook, CA, USA), respectively. Rats were then given 2 g/kg body weight as glucose solution by oral gavage. The tail blood samples were taken at 30, 60, 90 and 120 min after glucose administration.

### 2.5. Estimation of Biochemical Analysis of Plasma

The levels of triglyceride (TG), blood urea nitrogen (BUN), total billiubin (T-bill), glutamic-oxaloacetic transaminase (GOT) and glutamic-pyruvic transaminase (GPT) in plasma were enzymatically measured using commercially available kits (ARKRAY, Inc., Minami-ku, Kyoto, Japan). Plasma total cholesterol, low density lipoprotein (LDL)-cholesterol and HDL-cholesterol were determined using HDL and LDL/very low density lipoprotein (VLDL) Assay kit (E2HL-100, BioAssay Systems, Hayward, CA, USA). The plasma concentration ofleptin and insulin were measured based on the ELISA method using commercial rat leptin and insulin ELISA kit (Leptin Rat ELISA ab100773, Abcam, Cambridge, MA, USA; Insulin, 80-INSRT-E01, ALPCO, Cambridge, MA, USA).

### 2.6. Protein Preparation and Immunoblotting in the Rat Aorta and Muscle

Thoracic aorta and muscle were homogenized in a buffer consisting of 250 mM sucrose, 1 mM EDTA, 0.1 mM phenyl methylsulfonyl fluoride, and 20 mM potassium phosphate buffer (pH 7.6). Large tissue debris and nuclear fragments were removed by two successive low-speed spins (3500 rpm, 5 min; 8000 rpm, 10 min, 4 °C). Quantity of protein was measured by the Bradford method. An equal amount (35 μg) of protein was separated by 10% Sodium Dodecyl Sulfate (SDS)-PAGE. After electrophosis, protein was transferred electrophoretically to nitrocellulose membranes using a Mini-Protean II apparatus (Bio-Rad, Hercules, CA, USA). The membranes were then blocked by 5% bovine serum albumin (BSA) powder in 0.05% Tween 20-Tris-bufferd saline (TBS-T) for 1 h, and subsequently washed and incubated with primary antibodies to VCAM-1, ICAM-1, E-selectin and ET-1 (in aorta) and Insulin receptor substrate-1 (IRS-1) and glucose transporter type 4 (Glut4) (in muscle) (Santa Cruz Biotechnology, Santa Cruz, CA, USA) at a final dilution of 1:1000 overnight at 4 °C. After washing with TBS-T, membranes were incubated with the appropriate horseradish peroxidase-conjugated secondary antibody for 1 h. Signals were detected by a chemiluminescence (ECL) using detection system (Amersham, Buchinghamshire, UK). The bands were analyzed densitometrically by using a Chemi-doc image analyzer (Bio-Rad, Hercules, CA, USA).

### 2.7. Histopathological and Oil Red OStaining of Aortic Tissues, Epididymal Fat and Liver Tissues

For histopathological staining, aortic tissues were fixed in 10% (*v/v*) formalin in 0.01 M phosphate buffered saline (PBS) for 2 days with a change of formalin solution every day to remove traces of blood from tissue. The tissue samples were embedded in paraffin, and then thin sections (6 μm) of the aortic arch in each group were cut and stained with hematoxylin and eosin (H & E) stain for histopathological comparisons. 

Epididymal fat and liver tissues were fixed by immersion in 4% paraformaldehyde for 2 days at 4 °C, and incubated with 30% sucrose for 2 days. Each fat and liver was embedded in an optimum cutting temperature (OCT) compound (Tissue-Tek, Sakura Finetek, Torrance, CA, USA), frozen in liquid nitrogen, and stored at −80 °C. Frozen sections were cut with a Shandon Cryotome Special Motorized Electronic (SME) (Thermo Electron Corporation, Pittsburg, PA, USA) and placed on poly-l-lysine-coated slide. Epididymal fat sections were stained with H & E. For quantitative histopathological comparisons, each section was determined by Axiovision 4 Imaging/Archiving software (Axiovision 4, Carl Zeiss, Oberkochen, Germany). Liver sections were assessed by using Oil red o staining. Each section was stained with Oil red O for 20 min at room temperature after rinsing with 60% isopropyl alcohol and distilled water. Images of Oil red O stained liver were taken with Axiovision 4 Imaging/Archiving software. For quantitative analysis, the average scores of 10–20 randomly selected areas were calculated by using National institutes of health (NIH) Image analysis software, Image J (NIH, Bethesda, MD, USA).

### 2.8. Immunihistochemical Staining of Aortic Tissues

Parraffin sections for immunohistochemical staining were placed on poly-l-lysine-coated slides (Fisher Scientific, Pittsburgh, PA, USA). Slides were immunostained by Invitrogen’s HISOTO-STAIN^®^-SP kits (Carlsbad, CA, USA) using the Labeled-[strept] Avidin-Biotin (LAB-SA) method. After antigen retrieval, slides were immersed in 3% hydrogen peroxide for 10 min to block endogenous peroxidase activity. After being rinsed with PBS, slides were incubated with 10% non-immune goat serum for 10 min and incubated with primary antibodies of ICAM-1, VCAM-1 and ET-1 (1:200; Santa Cruz, CA, USA) in humidified chambers overnight at 4 °C. All slides were then incubated with biotinylated secondary antibody for 20 min, and then incubated with horseradish peroxidase-conjugated streptavidin for 20 min. Peroxidase activity was visualized by 3,3′-Diaminobenzidine (DAB; Novex^®^, Los Angeles, CA, USA) substrate-chromogen system, and counterstaining with hematoxylin (Zymed, Carlsbad, CA, USA). For quantitative analysis, the average scores of 10–20 randomly selected areas were calculated by using NIH Image analysis software, Image J (NIH, Bethesda, MD, USA).

### 2.9. Statistical Analysis

All the experiments were repeated at least three times. The results were expressed as a mean ± S.E., and the data were analyzed using one-way ANOVA followed by a Dunnett’s test or Student’s *t*-test to determine any significant differences. *p* < 0.05 was considered as statistically significant.

## 3. Results

### 3.1. Effects of FRG on Changes in Body Weight, Liver Weight and Epididymal Fat Pad Weight

Rats from all five groups showed significant increases in body weight gain during the experimental period. There was no significant change in body weight after eight weeks of high-fructose feeding in HF diet groups compared with the control group. However, treatment of FRG and losartan (Los.) groups showed significant decreases in body weight (Table 1). There was no significant change in food intake in all groups. Although there were no significant changes in body weight between HF groups and RG groups, there were decrease those levels. Moreover, HF diet resulted in a significant increase in liver weight and epididymal fat pad weight. Liver weight and epididymal fat pad weight were 34.81% and 40.92% higher than that of the HF diet group compared with the control group, respectively. However, treatment of the FRG group significantly reduced the liver weight and epididymal fat pad weight (23.44%, 38.26%) compared with HF diet groups, respectively. Similarly, treatment of losartan showed similar results to the FRG group. Although there was no significant change in liver weight between HF groups and RG groups, they decrease at those levels (Table 1).

### 3.2. Effect of FRG on the Morphology of Epididymal Fat Pads

**Figure 1:**
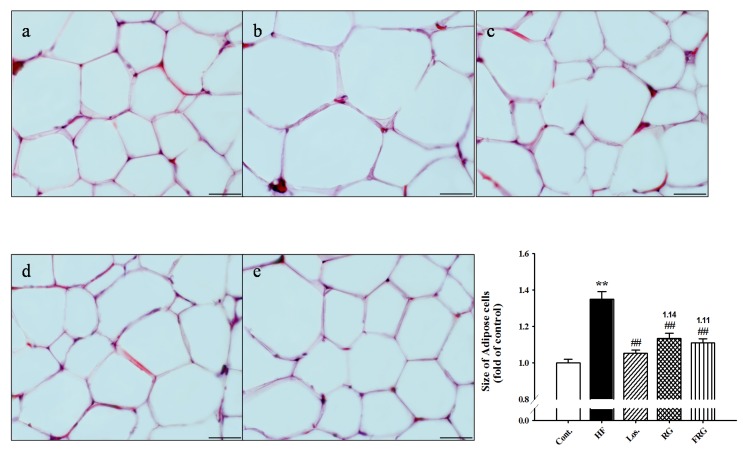
Effects of fermented red ginseng (FRG) on adipocytes on high fructose (HF) diet rats. Representative microscopic photographs of hematoxylin and eosin (H & E) stained sections of epididymal fat pads in HF diet rats. The lower panels indicated the size of adipose cells (magnification ×400). Scale bar shows 50 μm. (**a**) control; (**b**) HF; (**c**) HF + Losartan (Los.); (**d**) HF + RG (red ginseng); (**e**) HF + FRG (fermented red ginseng). Values were expressed as mean ± S.E. (*n* = 3). ** *p* < 0.01 *versus* Cont.; ^##^ *p* < 0.01 *versus* HF.

Because FRG effectively reduced the epididymal fat pad weight, we prepared frozen sections of epididymal fat pads and stained them with H & E. Histological findings, as shown in Figure 1, revealed hypertrophy of adipocytes in HF diet groups compared with the control group (+34.86%, *p* < 0.01). However, treatment of FRG, Los. and RG groups showed significantly decreases in the hypertrophy of adipocytes (−23.81%, −29.62% and −21.44% respectively, *p* < 0.01) (Figure 1). 

### 3.3. Effect of FRG on Plasma Lipid Levels

After eight weeks of fructose feeding, rats of HF diet groups showed a significant increase in plasma triglycerides, total cholesterol, and LDL-cholesterol levels compared with the control group. However, biochemical analysis of blood samples of the FRG groups showed significant decreases of T-Cho (104.2 ± 11.5 *versus* 70.3 ± 7.1 mg/dL, *p* < 0.05) and LDL-c (30.0 ± 2.6 *versus* 22.2 ± 1.7 mg/dL, *p* < 0.05) when compared with HF diet groups (Table 2), respectively.Although there was no significant change in triglyceride levels between HF group and FRG groups, there tended to decrease at those levels. Similarly, Los. group, TG, T-Cho and LDL-c were significantly lower than those levels of the HF diet group. Moreover, FRG was found to be more effective in reducing the elevated plasma triglycerides, total cholesterol, and LDL-cholesterol levels compared with RG groups. Besides the plasma levels of HDL-c levels in administration of Los., RG and FRG groups increased compared with HF diet groups (45.2 ± 4.1 *versus* 56.4 ± 1.6, 56.0 ± 3.1 and 60.5 ± 4.4 mg/dL, respectively, *p* < 0.05) (Table 2).

### 3.4. Effect of FRG on Plasma Parameters

Although there was no significant change of glutamic oxaloacetic transaminase (GOT), glutamic pyruvic transaminase (GPT), T-bills and non-fasting blood glucose levels in HF groups and administration drug groups, they decrease at those levels (Table 3). Similar to the plasma lipid levels, the plasma levels of leptin and insulin were significantly increased in HF diet groups compared with the control group. However, treatment of drug groups, especially FRG and Los. Groups, showed a significantly decreased in those levels compared with HF diet groups (Table 2). Moreover, FRG was found to be more effective in reducing the elevated plasma insulin level compared with RG groups.

### 3.5. Effect of FRG on Oral Glucose Tolerance Tests

**Figure 2:**
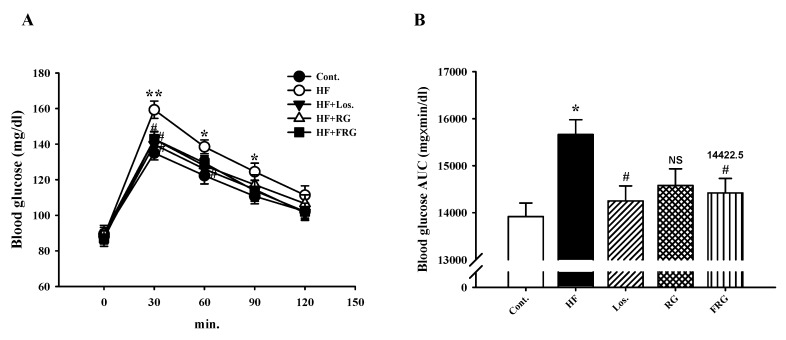
Effect of an FRG on oral glucose tolerance tests (**A**) and the blood glucose area under curve (AUC) (**B**). Values were expressed as mean ± S.E. (*n* = 10). * *p* < 0.05, ** *p* < 0.01 *versus* Cont.; ^#^ *p* < 0.05 *versus* HF. Abbreviations: HF, high fructose; HF + Los., high fructose diet with losartan; HF + RG, high fructose diet with red ginseng; HF + FRG, high fructose diet with fermented red ginseng.

Oral glucose tolerance tests were carried out to check insulin resistance in high-fructose diet rats after eight weeks. The results showed that HF diet groups maintained a significant increase in blood glucose levels at 30 (*p* < 0.01), 60 and 90 min (*p* < 0.05), respectively. However, the plasma glucose levels in treatment of FRG and RG groups were significantly decreased at 30 min as compared with HF diet groups (*p* < 0.05) (Figure 2A). Similarly, treatment of Los. groups was significantly decreased at 30 and 60 min as compared with HF diet groups. Moreover, area analysis (AUC) showed that HF diet groups significantly increased compared with the control group. However, FRG and Los. groups was significantly decreased compared to that of the HF groups (Figure 2B). Moreover, FRG was found to be more effective in reducing the elevated plasma glucose level compared with RG group.

### 3.6. Effect of FRG on Blood Pressure

**Figure 3:**
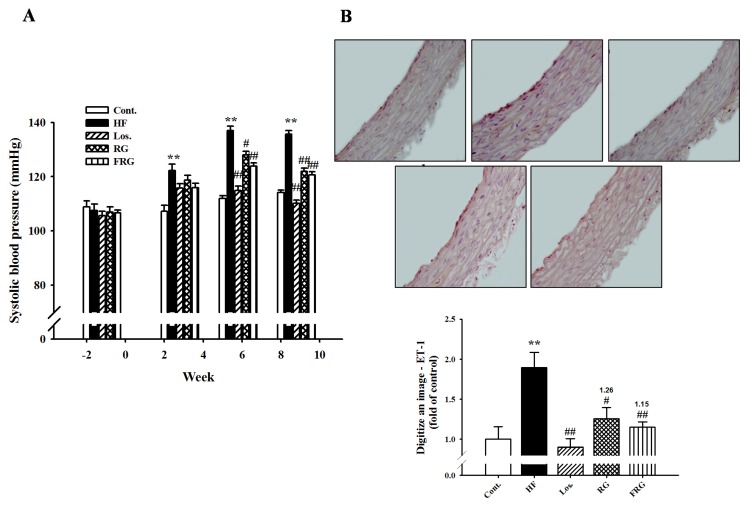
Effects of FRG on systolic blood pressure (**A**) and expression of ET-1 immunoreactivity (**B**) in aortic tissues of HF diet rats. Representative immunohistochemistry and quantifications are shown. Values were expressed as mean ± S.E. *n* = 10 (**A**) and *n* = 3 (**B**). ** *p* < 0.01 *versus* Cont.; ^#^ *p* < 0.05, ^##^ *p* < 0.01 *versus* HF.

**Figure 4:**
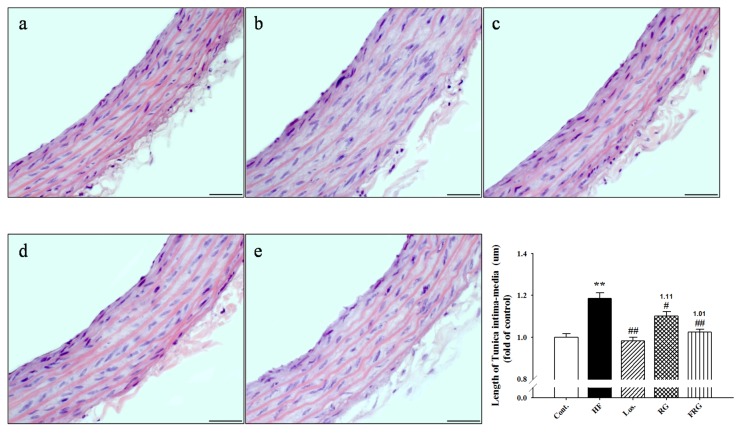
Representative microscopic photographs of H & E stained section of the thoracic aorta in HF diet rats. Lower panel indicated length of intima-media. Lower panel indicated the size of adipose cells (magnification ×400). (**a**) control; (**b**) HF; (**c**) HF + Los.; (**d**) HF + RG; (**e**) HF + FRG. Values were expressed as mean ± S.E. (*n* = 3). ** *p* < 0.01 *versus* Cont.; ^##^ *p* < 0.01 *versus* HF.

At the beginning of the experimental feeding period, the levels of systolic blood pressure in all groups were approximately 106–108 mmHg as investigated by the tail-cuff technique. After eight weeks, systolic blood pressure of HF groups were significantly increased compared to that of the control groups (*p* < 0.01). However, treatment of FRG and RG groups was significantly decreased compared to that of the HF groups during all experimental period (135.7 ± 1.3 *versus* 120.7 ± 1.1 and 122.0 ± 1.1, respectively, *p* < 0.01) (Figure 3A). Similarly, treatment of losartan showed similar results to FRG and RG groups. In addition, the protein expression of ET-1 level was increased in the HF diet group compared with the control group. However, administration drug groups showed significantly decreased expression levels of protein compared with HF diet groups (Figure 3B and Figure 4).

### 3.7. Effect of FRG on the Morphology of Aortas

**Figure 5:**
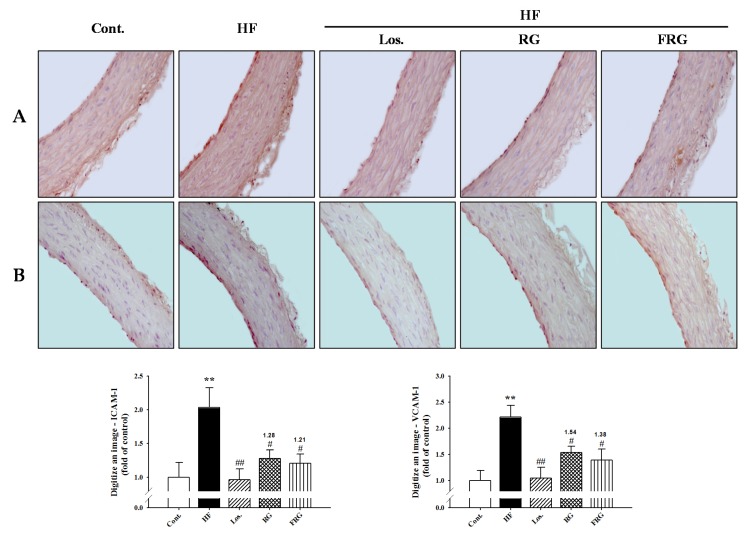
Effects of FRG on ICAM-1 (**A**) and VCAM-1 (**B**) immunoreactivity in aortic tissues of HF diet rats. Representative immunohistochemistry and quantifications are shown (magnification ×400. Values were expressed as mean ± S.E. (*n* = 3). ** *p* < 0.01 *versus* Cont.; ^#^ *p* < 0.05, ^##^ *p* < 0.01 *versus* HF. Abbreviations: HF, high fructose; HF + Los., high fructose diet with losartan; HF + RG, high fructose diet with Red ginseng; HF + FRG, high fructose diet with fermented Red ginseng.

FRG effectively decreased blood pressure. Thus, we examined staining with hematoxilin-eosin in thoracic aortas. Figure 5 showed that thoracic aortas of HF diet groups revealed roughened endothelial layers and increased tunica intima-media of layers compared with the control group. However, treatment of FRG and RG groups significantly maintained the smooth character of the intima endothelial layers and decreased tunica intima-media thickness in aortic sections. Similarly, treatment of losartan showed similar results to FRG and RG groups. Moreover, FRG was found to be more effective in maintaining the smooth character of the intima endothelial layers and decreased tunica intima-media thickness compared with RG groups.

### 3.8. Effect of FRG on the Expressions Levels of Adhesion Molecules and ET-1 in Aortas

**Figure 6:**
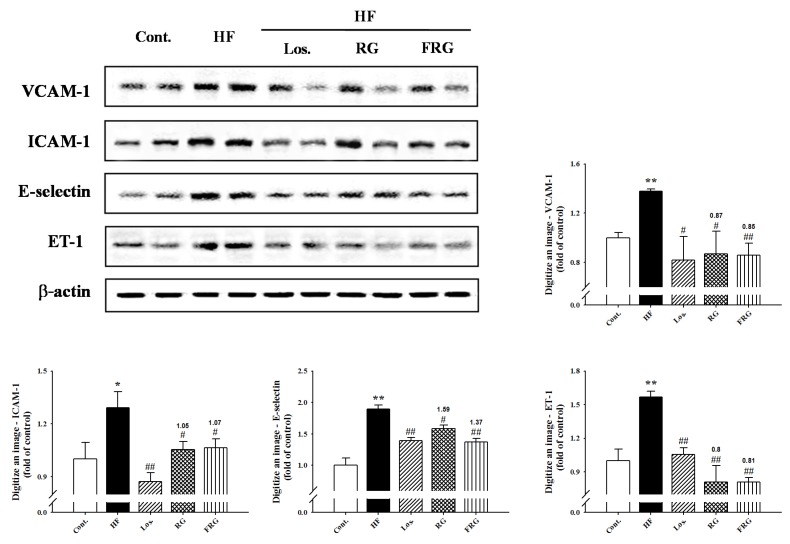
Effects of FRG on VCAM-1, ICAM-1, E-selectinm and ET-1 immunoreactivity in aortic tissues. Representative western blots of VCAM-1, ICAM-1, E-selectinm and ET-1 protein levels are quantifications are shown. Values were expressed as mean ± S.E. (*n* = 3). ** *p* < 0.01 *versus* Cont.; ^#^ *p* < 0.05, ^##^ *p* < 0.01 *versus* HF.

The protein expression of VCAM-1, ICAM-1, E-selectin and ET-1 in the descending aortas of all groups of rats was examined by Western blot analysis. Expression of adhesion molecules (VCAM-1, ICAM-1 and E-selectin) and ET-1 levels were increased in the HF diet groups compared with the control group. However, treatment of FRG and RG groups showed significantly decreased expression levels of protein compared with HF diet groups (Figure 6). Similarly, treatment of losartan groups showed similar results to FRG and RG groups.

Immunohistochemistry was performed to determine the direct expression of adhesion molecules in the aortic wall. Adhesion molecule expressions such as ICAM-1 and VCAM-1 were increased in the HF diet groups (*p* < 0.01). However, treatment of FRG and RG groups was significantly decreased expression levels of protein (ICAM-1 and VCAM-1, *p* < 0.05) (Figure 4). Similarly, treatment of losartan groups showed similar results to FRG and RG groups. Moreover, FRG was found to be more effective in decreased expression levels of protein levels compared with RG groups.

### 3.9. Effect of FRG on Hepatic Lipids

**Figure 7:**
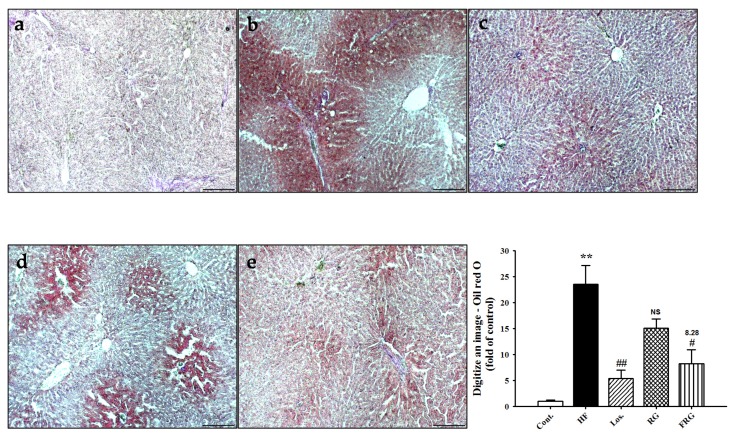
Representative microscopic photographs of Oil red O stained sections of the livers in HF diet rats. Representative Oil red O staining and quantifications are shown (magnification ×100). Values were expressed as mean ± S.E. (*n* = 3). ** *p* < 0.01 *versus* Cont.; ^#^ *p* < 0.05, ^##^ *p* < 0.01 *versus* HF. (**a**) control; (**b**) HF; (**c**) HF + Los.; (**d**) HF + RG (red ginseng); (**e**) HF + FRG (fermented red ginseng).

To investigate the existence of fat accumulation in the livers in all experimental groups, we prepared frozen sections of livers and stained them with Oil red O. Lipid droplets were detected in HF diet groups. However, treatment of FRG groups showed that the number of lipid droplets significantly decreased compared with HF diet groups (Figure 7). Similarly, treatment of losartan group showed similar results to the FRG groups. However, RG groups was no significant in RG groups compared with HF group. These results also showed that FRG was found to be more effective in decreased lipid droplets compared with RG groups.

### 3.10. Effect of FRG on the Expressions Levels of IRS-1 and Glut4in Muscle Tissue

**Figure 8:**
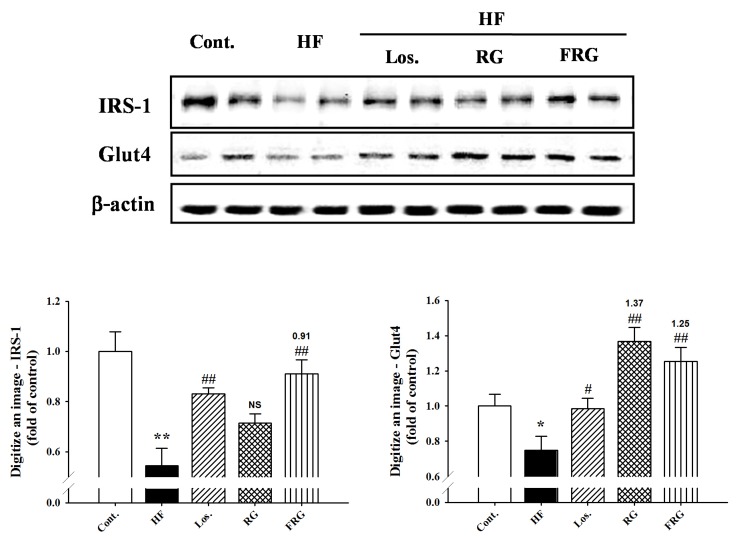
Effect of FRG on the expression of IRS-1 and Glut4 in the muscle of HF diet rats. Each electrophoretogram is representative of the results from three individual experiments. Values were expressed as mean ± S.E. (*n* = 3). ** *p* < 0.01 *versus* Cont.; ^##^ *p* < 0.01 *versus* HF. Abbreviations: HF, high fructose; HF + Los., high fructose diet with losartan; HF + RG, high fructose diet with red ginseng; HF + FRG, high fructose diet with fermented red ginseng.

To investigate the signals in insulin signaling, we examined the expression of IRS-1 and Glut4 in muscle tissue. The expression of IRS-1 and Glut4 were significantly decreased in HF diet groups. However, treatment of FRG groups increased expression levels of protein compared with HF diet groups (Figure 8). Similarly, treatment of losartan groups showed similar results to FRG groups.

## 4. Discussion

Past several years, many various plant extracts have been clinically evaluated for the treatment of metabolic disorder disease, such as diabetes, hypertension, dyslipidemia and atherosclerosis [24]. RG has been shown to possess beneficial clinical activity *in vivo* and *in vitro* studies [25,26]. Interestingly, the pharmacological effects of RG were further increased by fermentation. It has been reported that during the fermentation, the chemical composition of ginsenosides are transformed into their readily absorbable and more potent deglycosylated forms. In addition, FRG contains a much higher concentration of total ginsenosides and metabolites [27]. Therefore, future study will be required to clarify the active compounds of FRG and their pharmacological relevance for treatment of metabolic syndrome. 

In the present study, we investigated anti-obesity, hypotension, hypolipidemia improved glucose tolerance and ameliorated fatty liver effects of FRG in HF diet rats for eight weeks, and the efficacies were compared to those of RG and losartan. The HF diet induced rodent model is a well-established model of induced hypertension, hypertriglyceridemia, obesity, impaired glucose tolerance, fatty liver and vascular remodeling [28,29]. As expected, the present study showed that the results marked hypertension (increased systolic blood pressure and protein levels of ET-1), obese states (increased body weight, fat weight, adipocyte size and plasma levels of leptin), hyperlipidemia (increased plasma total cholesterol, triglycerides and LDL-cholesterol), impaired glucose tolerance (decreased protein levels of IRS-1 and Glut4), fatty liver (increased liver weight and fat accumulation) and vascular remodeling (increased endothelial dysfunction and adhesion molecules) through eight weeks of an HF diet. 

It is reported that a high fructose diet induced high blood pressure, intima-media thickness of the aorta and progress of blood vessel inflammation, which is correlated with prognosis and extension of the initial stage of atherosclerosis and initial stage of cardiovascular disease [30,31]. In addition, endothelial dysfunction was also related with lipid metabolism disorders [32]. The present results showed that an HF diet induced hypertension, dyslipidemia and endothelial dysfunction. However, administering FRG improved endothelial dysfunction with the amelioration of hypertension and dyslipidemia. In addition, interestingly, more favorable anti-hypertension, anti-vascular, inflammatory and regulating lipid homeostasis effects were observed with FRG as compared with an equal dose of RG.

The accumulation of fat deposition and increase in adipocyte size in the body is a major characteristic of obesity, as well as an increase of leptin level processes of leptin resistance, which classically has been characterized by expansion of intra-adipose tissue [33]. Although some studies have reported that losartan has no anti-obesity effects [34], the present results shows losartan has anti-obesity effects that decreased body weight, epididymal fat pad weight, adipocyte size and induced development of leptin resistance [35,36]. Further study is required to clarify the regulatory mechanisms of obesity and leptin levels in metabolic syndrome models. In addition, FRG effectively inhibited the leptin resistance with the amelioration of downregulation of body weight, epididymal fat pad weight and adipocyte size. These results suggest that FRG may be useful for inhibiting the development of leptin resistance and obesity. In addition, FRG was observed to homeostasis about anti-obesity and regulating leptin..

Recently, report has been found a low activation state of AMP-activated protein kinase (AMPK) with metabolic disorder associated with impaired insulin sensitivity, fat accumulation and dyslipidemia [37,38]. It is suggested that fructose-driven leptin resistance in the present study may be associated with impaired leptin-mediated decrease in AMPK phosphorylation. Although we did not examine specific research related to energy metabolism in the AMPK pathway, we speculate that treatment of FRG could be related to the improvements of metabolic disorders by activation of AMPK-related signal pathways.

It is reported that a high fructose diet induced impaired glucose tolerance via the elevation of plasma triglyceride levels, which plays an important role in the development of such abnormalities as insulin resistance, type 2 diabetes, dyslipidemia and fatty liver [39,40]. In addition, there are two signal transduction pathways for glucose/insulin transport in skeletal muscles. IRS-1 is an important tool for stimulating glucose transport induced by insulin [41]. The magnitude of a dense membrane compartment of Glut4 is related to the degree of insulin stimulated and thereby improves glucose uptake in skeletal muscles [42]. Thus, these factors are important key elements in insulin-dependent signal transduction pathways [43]. The present results showed that an HF diet impaired glucose tolerance, which is downregulation of IRS-1 and Glut4 expression. Moreover, an HF diet produced increased liver weight and accumulation of fat deposition in the liver, whereas, FRG improved impaired glucose tolerance with upregulation of IRS-1 and Glut4 protein level expression and decreased liver weight and accumulation of fat deposition in the liver. Hepatic steatosis is associated with metabolic syndrome and is a risk factor for the development of chronic hepatitis. Hyperinsulinemia and hyperglycemia associated with various metabolic disorders may act directly to promote hepatic injury including inflammation and fatty liver. In addition to investigating damage of the liver with aggravation of inflammation, the present study measured plasma levels of GPT, GOT and T-bill. Although all groups did not observe significant changes in plasma levels of GPT, GOT and T-bill, FRG tended to ameliorate those levels. Taken together, the present results showed direct/indirect evidence that FRG has regulated insulin signals and hepatoprotective effects. Moreover, FRG was observed to have more favorable regulating insulin signals and hepatoprotective effects compared with equal doses of RG.

In the future, we plan for more studies that clarify specific mechanism research in relation to other energy metabolic controls in an intestinal body and what ingredients or compounds are most effective in fermented red ginseng in HF diet induced metabolic syndrome.

## 5. Conclusions

Oral treatment of fermented red ginseng (FRG) effectively ameliorated HF diet induced metabolic disorders such as obesity, dyslipidemia, hypertension and fatty liver. Moreover, treatment of FRG had more favorable pharmacological effects on HF diet induced metabolic disorders compared with an equal dose of red ginseng. Taken together, fermented red ginseng might be a beneficial therapeutic approach for metabolic syndrome.

## Figures and Tables

**Table 1:** Effect of fermented red ginseng FRG on body weight, liver weight and epididymal fat pads.

Groups	Control	HF	HF		
			Los.	RG	FRG
Initial BW (g)	270.5 ± 3.3	270.0 ± 3.1	275.8 ± 3.6	272.6 ± 1.8	275.5 ± 4.3
Terminal BW (g)	399.6 ± 7.4	421.2 ± 6.0	384.1 ± 9.8 ^#^	405.8 ± 10.5	393.9 ± 6.4 ^#^
Food intake (g/day)	18.5 ± 0.3	18.4 ± 0.4	18.2 ± 0.5	18.8 ± 0.4	18.4 ± 0.5
Liver weight (g)	9.0 ± 1.3	12.3 ± 0.4 *	9.2 ± 0.4 ^##^	10.5 ± 0.7	9.9 ± 0.3 ^#^
Epididymal fat pads weight (g)	6.4 ± 0.9	9.0 ± 0.4 *	5.5 ± 0.4 ^##^	7.6 ± 0.7 ^#^	6.6 ± 0.7 ^#^

Values were expressed as mean ± S.E. (*n* = 10). * *p* < 0.05 *versus* Cont.; ^#^ *p* < 0.05, ^##^ *p* < 0.01 *versus* HF. Abbreviations: HF, high fructose; HF + Los., high fructose diet with losartan; HF + RG, high fructose diet with red ginseng; HF + FRG, high fructose diet with fermented red ginseng; BW, body weight.

**Table 2:** Effect of FRG on plasma lipids.

Groups	Control	HF	HF		
			Los.	RG	FRG
T-Cho (mg/dL)	69.1 ± 7.2	104.2 ± 11.5 *	72.5 ± 5.5 ^#^	80.0 ± 4.1 ^#^	70.3 ± 7.1 ^#^
TG (mg/dL)	155.0 ± 12.8	243.6 ± 27.5 *	117.8 ± 17.1 ^##^	180.3 ± 32.6	169.0 ± 25.1
HDL-c (mg/dL)	57.7 ± 5.5	45.2 ± 4.1	56.4 ± 1.6 ^#^	56.0 ± 3.1 ^#^	60.5 ± 4.4 ^#^
LDL-c (mg/dL)	22.2 ± 2.1	30.0 ± 2.6*	20.2 ± 1.8 ^#^	26.0 ± 1.6	22.2 ± 1.7 ^#^

Values were expressed as mean ± S.E. (*n* = 10). * *p* < 0.05 *versus* Cont.; ^#^ *p* < 0.05 *versus* HF. Abbreviations: HF, high fructose; HF + Los., high fructose diet with losartan; HF + RG, high fructose diet with red ginseng; HF + FRG, high fructose diet with fermented red ginseng; T-Cho, total cholesterol; TG, triglyceride; HDL-c, high-density lipoprotein cholesterol; LDL-c, low-density lipoprotein cholesterol.

**Table 3:** Effect of FRG treatment on plasma parameters.

Groups	Control	HF	HF		
			Los.	RG	FRG
GOT (IU/L)	137.9 ± 10.5	164.3 ± 15.0	155.1 ± 9.8	161.8 ± 28.6	137.00 ± 16.4
GPT (IU/L)	22.2 ± 3.8	29.3 ± 6.7	30.3 ± 4.5	26.8 ± 1.8	28.1 ± 6.5
T-bill (mg/mL)	0.49 ± 0.04	0.48 ± 0.04	0.48 ± 0.03	0.49 ± 0.02	0.38 ± 0.03
Leptin (ng/mL)	5.76 ± 1.0	10.93 ± 2.0 *	3.80 ± 0.7 ^##^	6.32 ± 1.1	5.41 ± 1.3 ^#^
Insulin (ng/mL)	2.1 ± 0.5	4.1 ± 0.6 *	2.0 ± 0.5 ^#^	2.4 ± 0.6	2.2 ± 0.3 ^#^
Blood glucose (mg/dL)	129.4 ± 5.6	136.9 ± 3.2	128.1 ± 4.6	129.4 ± 7.1	130.3 ± 2.9

Values were expressed as mean ± S.E. (*n* = 10). * *p* < 0.05 *versus* Cont.; ^#^ *p* < 0.05 *versus* HF. Abbreviations: HF, high fructose; HF + Los., high fructose diet with losartan; HF + RG, high fructose diet with red ginseng; HF + FRG, high fructose diet with fermented red ginseng; GOT, glutamic-oxaloacetic transaminase; GPT, glutamic-pyruvic transaminase; T-bill, total billiubin.

## Supplementary Materials

The following are available online at http://www.mdpi.com/2072-6643/8/6/369/s1, Table S1: Composition of diet obtained from Research diet.
