# Supplementary material for: Fermented Red Ginseng Potentiates Improvement of Metabolic Dysfunction in Metabolic Syndrome Rat Models
Source: Nutrients. 2016 Jun 16;8(6):369. doi: 10.3390/nu8060369 (PMC4924210; doi:10.3390/nu8060369)
Supplement: Supplementary file 1 [file nutrients-08-00369-s001.docx]

Supplementary Materials: Fermented Red Ginseng Potentiates Improvement of Metabolic Dysfunction in Metabolic Syndrome Rat Models

Min Chul Kho, Yun Jung Lee, Ji Hun Park, Hye Yoom Kim, Jung Joo Yoon, You Mee Ahn, Rui Tan, Min Cheol Park, Jeong Dan Cha, Kyung Min Choi, Dae Gill Kang and Ho Sub Lee

**Table S1.** Composition of diet obtained from Research diet.

| **Product #** | **D12450B #  (Rodent Diet with 10 kcal% Fat)** | | **D02022704  (Rodent Diet with 70 kcal% Fructose)** | |
| --- | --- | --- | --- | --- |
|  | gm% | kcal% | gm% | kcal% |
| Protein | 19.2 | 20.0 | 19.2 | 20.0 |
| Carbohydrate | 67.3 | 70.0 | 67.3 | 70.0 |
| Starch | 33.2 | 34.5 | 8.5 | 8.9 |
| Sucrose | 34.1 | 35.5 | 0.9 | 1.0 |
| Fructose | 0.0 | 0.0 | 57.8 | 60.1 |
| Fat | 4.3 | 10.0 | 4.3 | 10.0 |
| Total |  | 100.0 |  | 100.0 |
| Kcal/gm | 3.85 |  | 3.85 |  |
| Ingredient | gm | kcal | gm | kcal |
| Casein, 80 Mesh | 200 | 800 | 200 | 800 |
| l-Cysine | 3 | 12 | 3 | 12 |
| Corn starch | 315 | 1260 | 90 | 36 |
| Maltodesirin 10 | 35 | 140 | 0 | 0 |
| Sucrose | 350 | 1400 | 0 | 0 |
| Fructose | 0 | 0 | 610 | 2440 |
| Cellurose, BW200 | 50 | 0 | 50 | 0 |
| Soybean Oil | 25 | 225 | 25 | 225 |
| Lard | 20 | 180 | 20 | 180 |
| Mineral Mix S10026 | 10 | 0 | 10 | 0 |
| Dicalcium phosphate | 13 | 0 | 13 | 0 |
| Calcium carbonate | 5.5 | 0 | 5.5 | 0 |
| Potassium Citrate, 1 H_2_O | 16.5 | 0 | 16.5 | 0 |
| Vitamin Mix V10001 | 10 | 40 | 10 | 40 |
| Choline Bitartrate | 2 | 0 | 2 | 0 |
| FD & C Yellow Dye #5 | 0.05 | 0 | 0 | 0 |
| FD & C Red Dye #40 | 0 | 0 | 0 | 0 |
| FD & C Blue Dye #1 | 0 | 0 | 0.05 | 0 |
| Total | 1055.05 | 4057 | 1055.05 | 4057 |
